# Effects of smoking and body mass index on the exposure of fentanyl in patients with cancer

**DOI:** 10.1371/journal.pone.0198289

**Published:** 2018-06-08

**Authors:** Evelien J. M. Kuip, Wendy H. Oldenmenger, Martine F. Thijs—Visser, Peter de Bruijn, Astrid W. Oosten, Esther Oomen—de Hoop, Stijn L. W. Koolen, Carin C. D. Van der Rijt, Ron H. J. Mathijssen

**Affiliations:** 1 Department of Medical Oncology, Erasmus MC Cancer Institute, Rotterdam, the Netherlands; 2 Department of Medical Oncology, Radboud University Medical Center, Nijmegen, the Netherlands; 3 Department of Anesthesiology, Pain and Palliative Care, Radboud University Medical Center, Nijmegen, the Netherlands; 4 Netherlands Comprehensive Cancer Organisation, Utrecht, the Netherlands; University of Ottawa, CANADA

## Abstract

The transdermal fentanyl patch is widely used to treat cancer-related pain despite its wide inter- and intrapatient variability in pharmacokinetics. The aim of this study was to investigate whether smoking and body size (i.e. body mass index) influence fentanyl exposure in patients with cancer. These are factors that typically change during treatment and disease trajectories. We performed an explorative cohort study in patients with cancer using transdermal fentanyl patches (Durogesic^®^), by taking a blood sample for pharmacokinetic analysis one day after applying a patch in patients with a stable fentanyl dose. A total of 88 patients were evaluable. Although no statistically significant difference was found, the plasma concentrations of non-smokers was 28% (95% CI [-14%; +89-%]) higher than those of smokers normalizing for a dose of 25μg/min. Patients with a low BMI (< 20 kg/m2) had almost similar (10% (95% CI [-39%; +97%]) higher) plasma concentrations compared to patients with a high BMI (> 25 kg/m2). A wider variation in fentanyl plasma concentrations was found in this study than anticipated. Due to this variation, studies in larger patient cohorts are needed to further investigate the effect of smoking on plasma concentration of fentanyl and thereby clarify the clinical significance of our findings.

## Introduction

Fentanyl is one of the most commonly used (strong acting) opioids to treat cancer-related pain [1–3] and it is often preferred over morphine, especially in patients with renal failure. Additionally, fentanyl usually results in less obstipation than other opioids [4–6]. The fentanyl transdermal patch has been used since decades to treat chronic pain. Fentanyl is also available in a liquid formulation for intravenous and subcutaneous administration and in various immediate release forms for oromucosal and nasal use [7, 8]. Fentanyl is highly lipophilic and is therefore rapidly absorbed by the subcutaneous fat-tissue. After placement of the patch fentanyl is absorbed by the skin. When the patch is removed systemic fentanyl concentration will slowly decrease as a result of fentanyl release from subcutaneous depots (formed below the patch) [7, 9, 10].

Fentanyl is mainly metabolized in the liver by cytochrome P450 (CYP) enzymes, primarily by the CYP3A4 isozyme into the pharmacologically inactive metabolite norfentanyl by N-dealkylation. Fentanyl is mostly excreted by the kidney of which approximately 10% is unchanged. A minor part is excreted through the feces. There is a wide inter- and intra-patient variability in fentanyl pharmacokinetics (PK). Several factors have been studied to clarify this variability in fentanyl pharmacokinetics; liver function, strong CYP3A4 inhibitors and inducers and adding local heat to transdermal patches evidently affect fentanyl pharmacokinetics but only partly explain the wide variability mentioned [7]. Other unknown factors may also be contributing to this variation.

Two patient characteristics that may change during various stages of cancer are body size measures and smoking habits, while their effects on fentanyl exposure in patients with cancer are currently unknown. The body mass index (BMI) is one of the most commonly used body size measures and is calculated by weight (in kilogram) divided by the square of height. In patients with advanced disease a decrease in BMI is common and this may lead to a changed body composition, changed thickness of the skin, and changed skin permeability. Some studies already studied the influence of BMI on fentanyl pharmacokinetics with contradictory results. One study reported that cachectic patients with cancer treated with transdermal fentanyl patches had significantly lower fentanyl plasma concentrations compared to normal weight patients [11]. Other studies did not find differences in fentanyl levels between normal weight and low weight patients with cancer [12, 13].

Smoking is a daily routine for many patients with cancer. If smoking alters fentanyl exposure, a change in smoking habits like (re-)starting or quitting smoking may influence fentanyl pharmacokinetics and therefore its effects on pain. The polycyclic aromatic hydrocarbons in cigarette smoke are believed to be responsible for the induction of CYP iso-enzymes [14, 15]. Cigarette smoking was shown to induce drug metabolism in patients using diazepam, haloperidol and/or caffeine [16–18]. Furthermore, smoking patients showed a lower exposure to erlotinib and irinotecan compared to non-smoking patients. Like fentanyl, erlotinib and irinotecan are also partly metabolized by CYP3A4 [19, 20]. Therefore, we hypothesized that smoking may have an effect on the exposure of fentanyl.

The aim of this prospective cohort study was to investigate the influence of BMI and smoking on the exposure of fentanyl in patients with cancer using a fentanyl patch.

## Methods

Our study was performed as a prospective single-center pharmacokinetic study at the Erasmus MC Cancer Institute. Patients were included from 1^st^ of April 2014 until 27^th^ of October 2015. Inclusion criteria were: patients with cancer ≥ 18 years, using a stable dose of a transdermal fentanyl (Durogesic^®^) for at least 8 days irrespective of the dose used and given written informed consent. Exclusion criteria were: use of fentanyl rescue medication (other opioids were allowed), the use of strong CYP inhibitors or inducers and serious psychiatric illness, confusion or intellectual disability. Smoking was defined as smoking tobacco daily. Non-smoking patients were defined as patients who had never smoked, or quit smoking at least one month before PK sampling. Patients were divided in three BMI groups; BMI <20 kg/m^2^ (low), BMI 20–25 kg/m^2^ (normal) and BMI >25 kg/m^2^ (high). Patients applied the patch to the upper arm. One venous blood sample was taken from the contralateral arm approximately 24 hours after application of a new patch. The blood samples were collected in potassium EDTA coated tubes. Patients were not evaluable when the appropriate blood samples were not taken. Lab results of levels of creatinine, estimated glomerular filtration rate (eGRF), calculated by Modification of Diet in Renal Disease (MDRD); [formula: eGFR (ml/min/1.73 m^2^) = 32788 x serum Creatinine (μmol/l) ^-1.154^ x age (years) ^-0.203^ x (0.742 when female) x (1.210 when of African descent)], aspartate aminotransferase (AST), alanine aminotransferase (ALT), bilirubin, albumin and alkaline phosphatase (ALP) were assessed. Protocol in S1 Protocol. Trend statement in S1 Trend.

The study was approved by the medical ethics review board of the Erasmus Medical Center (MEC 13.412) on the 27^th^ of March 2014 and conducted in accordance with the Declaration of Helsinki. Written informed consent was obtained from all patients. The trial was registered in the Dutch Trial Register (Trial registration ID: NTR4672) in July 2014. The authors confirm that all ongoing and related trials for this drug/intervention are registered.

### Measurements of fentanyl plasma concentrations

Fentanyl in plasma was quantitated using a validated UPLC-MS/MS method consisting of a Waters Acquity UPLC sample manager coupled to a triple quadruple mass spectrometer operating in the multiple reaction monitoring mode (MRM) with positive ion electrospray ionization (Waters, Etten-Leur, The Netherlands). The multiple reaction monitoring transition was set at 337→188.

Chromatographic separation of fentanyl was achieved on an Acquity UPLC^®^ BEH C18 1.7 μm 2.1 x 100 mm column eluted at a flow-rate of 0.350 mL/min on a gradient of methanol. The overall cycle time of the method was 6 minutes. The calibration curves were linear over the range of 0.1 to 10 ng/mL with the lower limit of quantitation validated at 0.1 ng/mL for fentanyl. The within and between-run precisions at five tested concentrations, including the LLQ, were ≤ 5.5% and ≤ 6.1%, respectively, while the average accuracy ranged from 86.2% to 97.5%. The extraction of 200 μL of plasma involved a deproteinization step with acetone followed by a simple liquid extraction with ethyl acetate. The organic phase was evaporated and subsequently dissolved in 100 μL methanolic solutions, from which aliquots of 10 μL were injected into the UPLC-MS/MS system.

### Statistics

For both the analysis on the effect of smoking and of BMI, a difference in exposure of fentanyl of 25% was judged clinically relevant. The inter-patient relative standard deviation in fentanyl pharmacokinetics was assumed to be 25%. Because two primary questions were to be answered, the Bonferroni correction was applied to account for multiple testing, resulting in a two-sided alpha of 0.025. Given a power of 80%, at least 20 patients were required in each BMI group (low versus high) to detect a difference based on the Student’s t-test. The assumption was that 25% of our population smoked. To include 20 smokers approximately 80 patients were needed in total. To compensate for non-evaluable patients (due to missing samples or other potential reasons) we aimed to include 100 patients.

As fentanyl has linear PK in the dose range used, doses were normalised to a dose of 25 μg/h for comparisons between patients [21, 22]. As the normalized plasma fentanyl concentrations turned out to follow a log-normal distribution, analyses were performed on the log-transformed concentrations and therefore, results will be presented as geometric means. These take into account the skewness of the data in contrast to arithmetic means which would have given unrealistic summary measures of the data. T-tests were performed on log-transformed data to compare 2 groups; smokers to non-smokers and low-BMI to high-BMI patients with respect to baseline characteristics. Bonferroni-corrected 95% confidence intervals were obtained by constructing 97.5% confidence intervals since the correction means that instead of alpha = 0.05 an alpha of 0.025 needs to be used.

## Results

**Fig 1:**
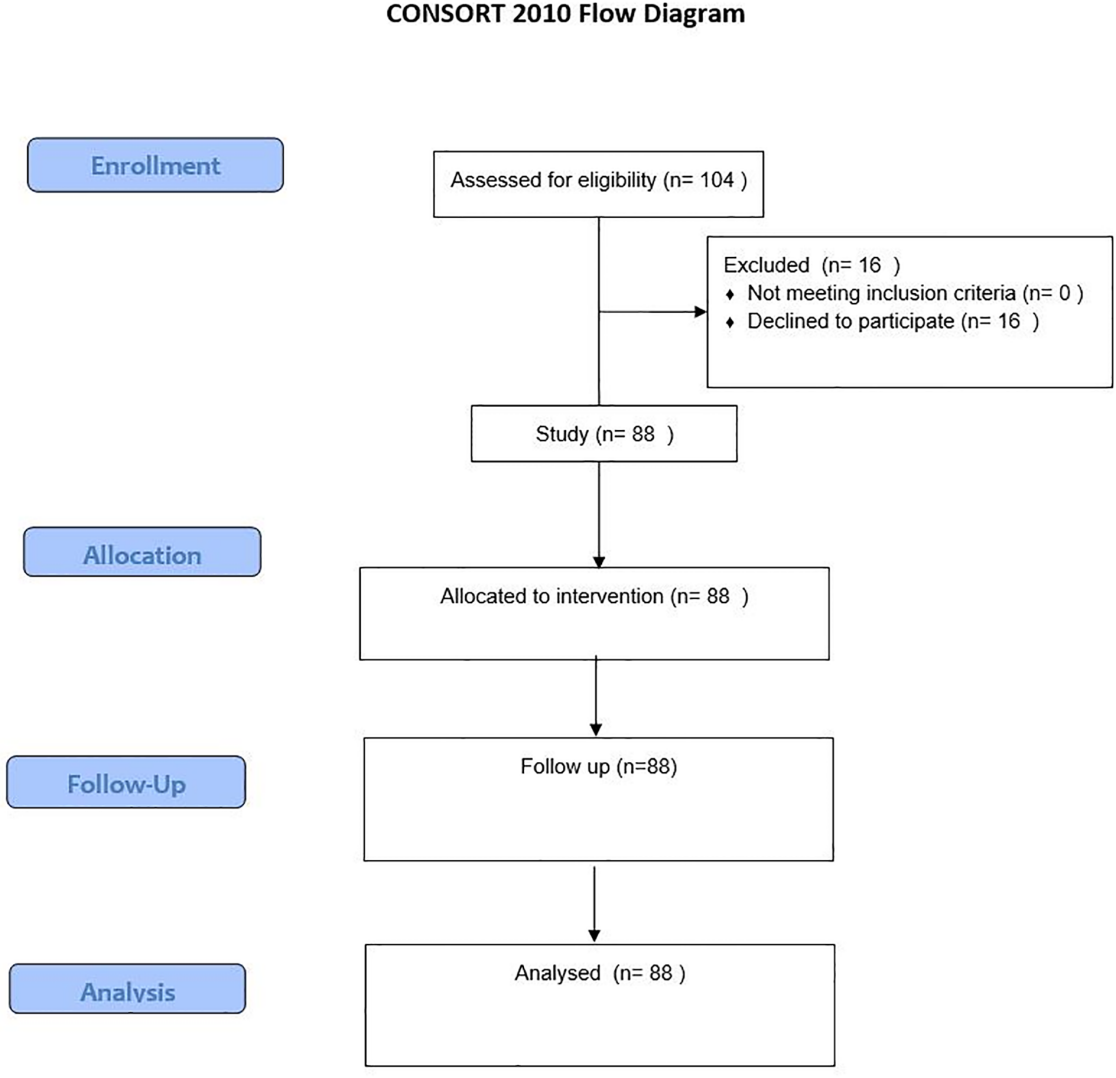
Consort diagram.

In total, 104 patients were included. Eighty-eight patients (39 males (44%) and 49 females (56%)) with a median age of 59.5 years (interquartile range (IQR) 53.5–67.0) completed the study and were evaluable, Fig 1. The demographic data of these evaluable patients are presented in Table 1. Twenty-seven patients (30.7%) were defined as smokers and 61 patients (69.3%) as non-smokers. In total, 20 patients had a BMI < 20 kg/m^2^ (22.7%), 41 patients had a BMI between 20–25 kg/m^2^ (46.6%) and 27 patients had a BMI > 25 kg/m^2^(30.7%). Creatinine, eGFR, AST, ALT, bilirubin, albumin and ALP turned out to follow a log-normal distribution. Only AST levels were significantly lower in smokers compared to non-smokers (p = 0.026), although this is unlikely to be clinically relevant. All patients had normal or limited (CTCAE grade 1) toxicities of creatinine, eGFR, AST, ALT, bilirubin, albumin or ALP.

**Table 1:** Patient characteristics.

Variable	Total n = 88	Smokers n = 27	Non smokers n = 61	BMI < 20 Median 18.5 n = 20	BMI 20–25 Median 23.0 n = 41	BMI > 25 Median 28.7 n = 27
**Sex, n(%)**						
- Male	39 (44)	15 (56)	24 (39)	13 (65)	16 (39)	10 (37)
- Female	49 (56)	12 (44)	37 (61)	7 (35)	25 (61)	17 (63)
**Age, years**						
median and IQR	60 (53–67)	59 (54–66)	60 (53–68)	60 (49–68)	59 (55–65)	62 (53–69)
**Height, cm**						
median and IQR	170 (163–178)	173 (165–181)	169 (163–177)	175 (169–184)	170 (163–178)	167 (163–177)
**Weight, kg**						
median and IQR	66 (60–78)	69 (62–78)	65 (59–78)	54 (51–63)	64 (61–73)	81 (74–96)
**Smoking (n, %)**	27 (31)	27 (100)	0 (0)	6 (30)	13 (32)	8 (30)
**Laboratory results** # (median (IQR)						
Creatinine^1^	66 (55–87)	70 (51–93)	65 (55–87)	59 (45–84)	62 (56–85)	78 (63–91)
MDRD^2^	88 (65–90)	89 (65–90)	87 (63–90)	90 (85–90)	90 (66–90)	71 (61–90)
AST^3^	27 (20–42)	22 (17–31)	30 (22–47)	22 (17–32)	28 (22–50)	31 (19–44)
ALT^4^	23 (13–34)	23 (13–27)	23 (15–40)	18 (14–27)	23 (13–34)	24 (13–45)
Bilirubin^5^	6 (4–8)	5 (4–8)	6 (4–8)	4 (3–7)	6 (4–8)	7 (5–9)
Albumin^6^	39 (36–43)	38 (35–42)	39 (37–44)	38 (32–42)	39 (36–43)	40 (37–44)
ALP^7^	114 (87–179)	93 (84–165)	124 (90–192)	108 (89–148)	118 (86–214)	111 (83–183)
**Fentanyl patch dose (μg/h)**						
median(IQR)	25 (12–50)	25 (12–50)	25 (12–50)	37 (15–50)	37 (12–69)	25 (12–25)

^#^ Normal ranges:

^1^ (55–90 umol/L);

^2^ (> 60 mL/min/1,73 m^2^);

^3^ (< 31 U/L);

^4^ (< 34 U/L);

^5^ (<17 umol/L);

^6^ (35–50 g/L);

^7^ (< 98 U/L)

**Fig 2:**
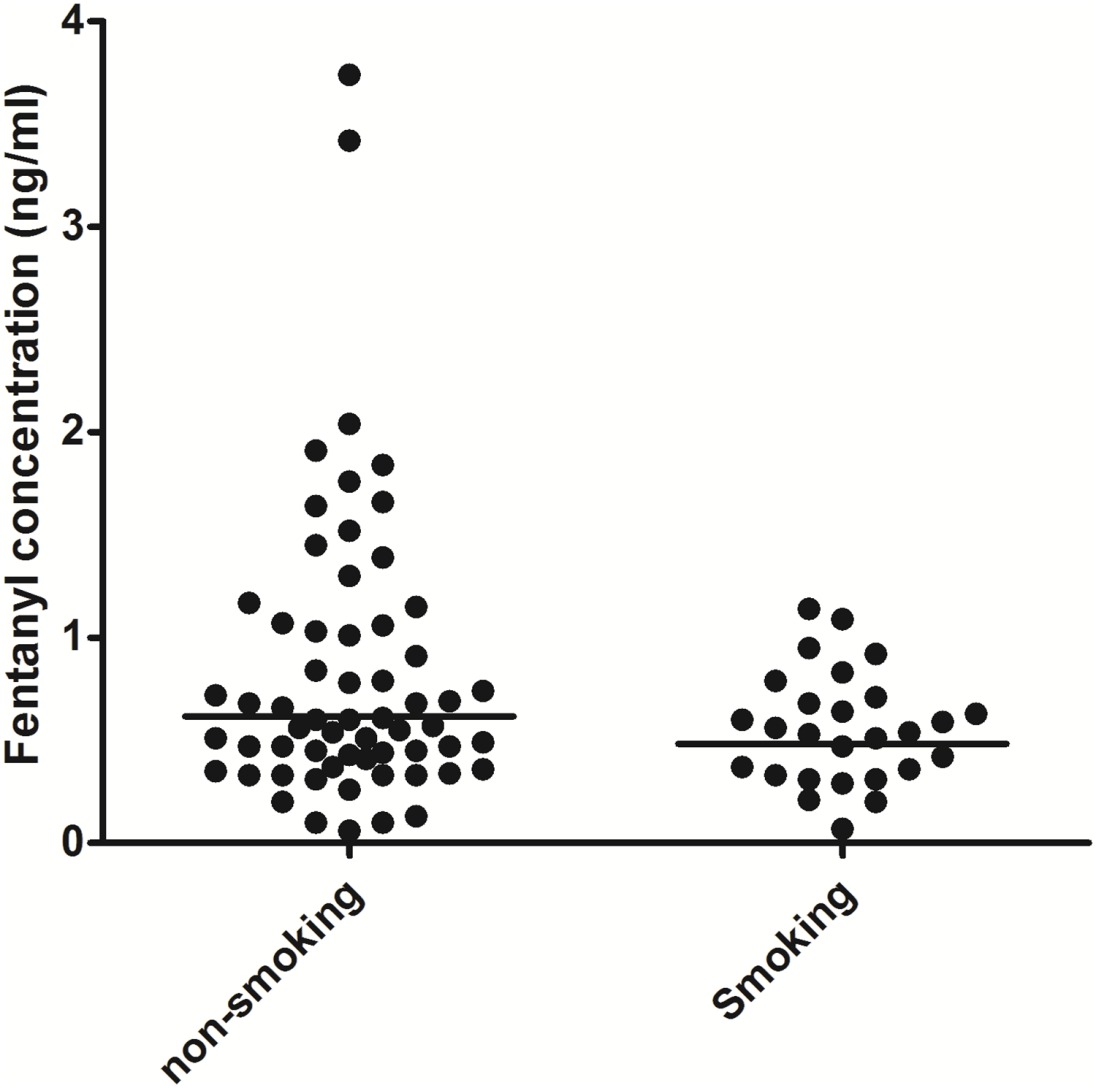
Normalized plasma fentanyl concentrations in patients; smokers and non-smokers. Horizontal line represents the median.

The normalized plasma concentrations of non-smokers and smokers were not statistically different (p = 0.32). The geometric means of the normalized plasma concentrations were 0.48 ng/ml (95% CI [0.38; 0.61]) for smokers and 0.62 ng/ml (95% CI [0.50; 0.76]) for non- smokers. The normalized plasma concentrations of non-smokers were 27.7% higher than those of smokers (95% CI [-13.8%; +89.1%], Fig 2).

**Fig 3:**
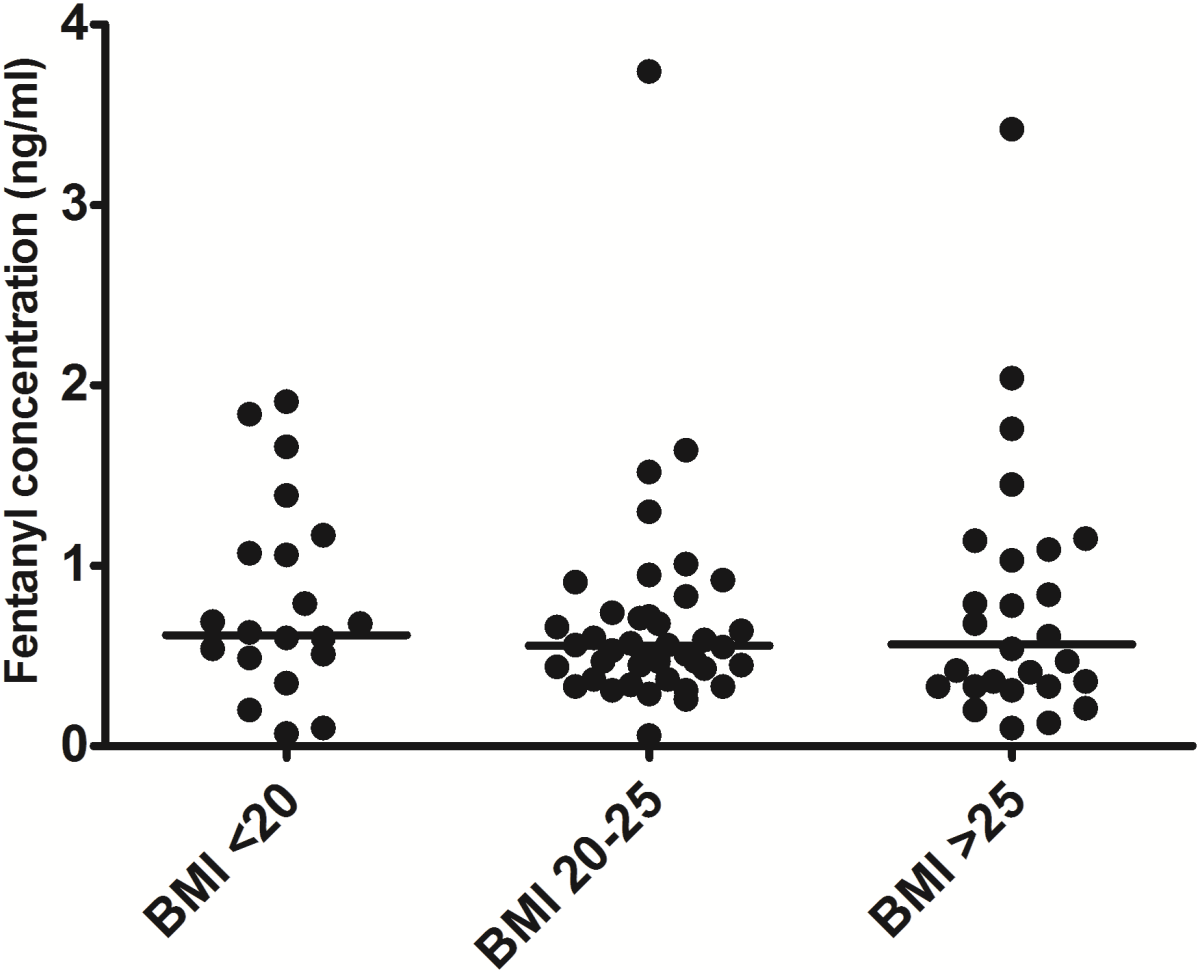
Normalized plasma fentanyl concentrations in patients; low, middle and high BMI patients. Horizontal line represents the median.

The normalized plasma concentrations of patients with a low or high BMI were also not statistically different (0.62 ng/ml (95% CI [0.41; 0.93]) vs 0.56 ng/ ml (95% CI [0.40; 0.79]), p = 1.00). Plasma concentrations of the low and high BMI groups did also not differ from the normal BMI group (0.56 ng/ml (95% CI [0.46; 0.68])). The concentration was 9.7% higher in patients with a low BMI as compared to patients with a high BMI (95% CI [-38.8%; 96.9%])Fig 3).

## Discussion

Interestingly, in this study, the interindividual variation in plasma fentanyl levels (geometric coefficient of variation = 87%) was much larger than our original assumption. Consequently, our study was underpowered to find a statistically significant difference in fentanyl plasma concentration. Nonetheless, based on our findings, the 27.7% higher normalized plasma concentrations of non-smokers compared to smokers, we cannot exclude an effect of smoking on fentanyl exposure. Smoking is a factor that frequently changes during phases of disease in patients with cancer. Together with the hypothesis that smoking leads to induction of CYP3A4 [20, 23] and that fentanyl metabolism might also be influenced by other (unknown) metabolic pathways [24, 25] it might be interesting to study smoking in a larger cohort of patients with cancer. Nevertheless, other strong inducers like rifampicin [26] and carbamazepine or phenobarbital [24] had highly relevant inductive effects on fentanyl PK. The combination of rifampicin and oral transmucosal fentanyl citrate led to a significant lower exposure to fentanyl compared to fentanyl alone (2.20 vs 5.87 ng/mL). A population pharmacokinetic analysis showed a significantly higher fentanyl clearance when patients used CYP3A4 inducers compared to patients who did not use CYP3A4 inducers [24].

Unfortunately our study lacks information about the daily consumption of cigarettes and its use over the years. Probably, the amount of cigarettes smoked daily influences the size of enzyme induction. All patients who were included in the non-smoking group did not smoke for at least one month before inclusion. The time to require maximum enzyme induction takes approximately two weeks [20, 23]. We therefore assume that potential late effects of enzyme induction by cigarette smoking were ruled out in this study since a minimum period of 4 weeks without smoking was required to be eligible for the non-smoking group.

Transdermal fentanyl is absorbed by the skin and a fentanyl depot concentrates in the upper skin layers. This is followed by uptake in the microcirculation and general circulation. Skin conditions of smokers are different compared to non-smokers; skin ageing is accelerated and leads to reduced functional capacity of the skin causing dryness of the skin and wrinkles [27–29]. This reduced skin condition might affect transdermal absorption of fentanyl. A limitation of this study is that we did not specify the skin conditions of the patients in this study and the sampling design did not allow us to describe the absorption phase. BMI did not significantly influence the exposure to transdermal fentanyl in our cohort of patients, which is in line with earlier studies [12, 13]. Hypoalbuminemia is common in patients with a low BMI and because of the lipophilic character of fentanyl, the binding of fentanyl to plasma proteins like albumin might be influenced by the plasma concentration of albumin [7, 12]. In our study, the albumin levels were within normal ranges and comparable in all BMI groups. The combination of low BMI and normal albumin did not influence fentanyl plasma concentration in another study [12]. Only a study by Heiskanen et al. showed significantly lower plasma fentanyl levels in cachectic patients with cancer compared to normal weight patients [11]. Probably this is due to the extremely low BMI group of < 16 in their study. An explanation for their finding might be lower skinfold thickness in cachectic patients. The thinner lipophilic part of the skin possibly impairs the absorption of lipophilic fentanyl [9]. Possibly, other factors, not studied, related to BMI and cachexia like CRP, skinfold and skin condition might also influence absorption.

In summary, we conducted a prospective cohort study to investigate the influence of smoking or BMI on plasma fentanyl concentrations. For both factors we did not find any evidence for an effect nor the lack of an effect. This study was underpowered because of unexpectedly large variations in fentanyl levels between patients. To study the effect of smoking and BMI on fentanyl levels a larger patient population needs to be tested.

## Supporting information

S1 Protocol Protocol BMI smoking. (DOC)

S1 Trend Trendstatement_TREND_Checklist BMI smoking.pdf. (PDF)
