# Supplementary material for: Effects of smoking and body mass index on the exposure of fentanyl in patients with cancer
Source: PLoS One. 2018 Jun 8;13(6):e0198289. doi: 10.1371/journal.pone.0198289 (PMC5993275; doi:10.1371/journal.pone.0198289)
Supplement: S1 Protocol — (DOC) [file pone.0198289.s001.doc]

Effects of body mass index (BMI) and smoking on the pharmacokinetics of fentanyl

**PROTOCOL TITLE**

**Effects of body mass index (BMI) and smoking on the pharmacokinetics of fentanyl.**

| **Protocol ID** | **MEC-2013-412** |
| --- | --- |
| **Short title** | **Factors influencing fentanyl PK** |
| **EudraCT number** | **not applicable** |
| **Version** | **3** |
| **Date** | **12-03-2014** |
| **Coordinating investigator/project leader** | **E.J.M. Kuip**  Department of Medical Oncology, Room G4-61 Erasmus MC Cancer Institute  Groene Hilledijk 301, 3075 EA Rotterdam, The Netherlands  Tel: +31 10 7041338  Fax: +31 10 7041003  Email: [e.kuip@erasmusmc.nl](mailto:e.kuip@erasmusmc.nl) |
| **Principal investigator(s) (in Dutch: hoofdonderzoeker/ uitvoerder)** | **Prof. Dr. R.H.J. Mathijssen**  Department of Medical Oncology, Room G4-80  Erasmus MC Cancer Institute  Groene Hilledijk 301, 3075 EA Rotterdam, The Netherlands  Tel: +31 10 7034897  Fax: +31 10 7041003  Email: [a.mathijssen@erasmusmc.nl](mailto:a.mathijssen@erasmusmc.nl) |
|  |  |
| **Sponsor (in Dutch: verrichter/opdrachtgever)** | **Prof. Dr. S. Sleijfer**  Department of Medical Oncology, Room G4-50  Erasmus MC Cancer Institute  Groene Hilledijk 301, 3075 EA Rotterdam, The Netherlands  Tel: +31 10 7041733  Fax: +31 10 7041003  Email: [s.sleijfer@erasmusmc.nl](mailto:c.vanderrijt@erasmusmc.nl) |
| **Laboratory sites** | **Laboratory of Translational Pharmacology, Erasmus MC**  Ir. P. De Bruijn |
|  |  |
| **Pharmacy** | **Poliklinische Apotheek Erasmus MC- Daniel den Hoed**  Groene Hilledijk 301  3075 EA Rotterdam |
|  |  |

**PROTOCOL SIGNATURE SHEET**

| **Name** | **Signature** | **Date** |
| --- | --- | --- |
| **Sponsor or legal representative:**  **Head of Department:** | **Prof. dr. S. Sleijfer**  Medical oncologist |  |
| **[Coordinating Investigator/Project leader/Principal Investigator]:** | **Prof. Dr. R.H.J. Mathijssen**  Department of Medical Oncology, Room G4-80  Erasmus MC Cancer Institute  Groene Hilledijk 301, 3075 EA Rotterdam, The Netherlands  Tel: +31 10 7034897  Fax: +31 10 7041003 |  |

**TABLE OF CONTENTS**

1. INTRODUCTION AND RATIONALE [9](#__RefHeading___Toc362990514)

2. OBJECTIVES [11](#__RefHeading___Toc362990515)

3. STUDY DESIGN [12](#__RefHeading___Toc362990516)

4. STUDY POPULATION [13](#__RefHeading___Toc362990517)

4.1. Population [13](#__RefHeading___Toc362990518)

4.2. Inclusion criteria [13](#__RefHeading___Toc362990519)

4.3. Exclusion criteria [13](#__RefHeading___Toc362990520)

4.4. Sample size calculation [13](#__RefHeading___Toc362990521)

5. TREATMENT OF SUBJECTS [14](#__RefHeading___Toc362990522)

5.1. Investigational product/treatment [14](#__RefHeading___Toc362990523)

5.2. Use of co-intervention (if applicable) [14](#__RefHeading___Toc362990524)

5.3. Escape medication (if applicable) [14](#__RefHeading___Toc362990525)

6. INVESTIGATIONAL PRODUCT [15](#__RefHeading___Toc362990526)

6.1. Name and description of investigational product(s) [15](#__RefHeading___Toc362990527)

6.2. Summary of findings from non-clinical studies [15](#__RefHeading___Toc362990528)

6.3. Summary of findings from clinical studies [15](#__RefHeading___Toc362990529)

6.4. Summary of known and potential risks and benefits [15](#__RefHeading___Toc362990530)

6.5. Description and justification of route of administration and dosage [15](#__RefHeading___Toc362990531)

6.6. Dosages, dosage modifications and method of administration [15](#__RefHeading___Toc362990532)

6.7. Preparation and labelling of Investigational Medicinal Product [15](#__RefHeading___Toc362990533)

6.8. Drug accountability [15](#__RefHeading___Toc362990534)

7. NON-INVESTIGATIONAL PRODUCT [16](#__RefHeading___Toc362990535)

7.1. Name and description of non-investigational product(s) [16](#__RefHeading___Toc362990536)

7.2. Summary of findings from non-clinical studies [16](#__RefHeading___Toc362990537)

7.3. Summary of findings from clinical studies [16](#__RefHeading___Toc362990538)

7.4. Summary of known and potential risks and benefits [16](#__RefHeading___Toc362990539)

7.5. Description and justification of route of administration and dosage [16](#__RefHeading___Toc362990540)

7.6. Dosages, dosage modifications and method of administration [16](#__RefHeading___Toc362990541)

7.7. Preparation and labelling of Non Investigational Medicinal Product [16](#__RefHeading___Toc362990542)

7.8. Drug accountability [16](#__RefHeading___Toc362990543)

8. METHODS [17](#__RefHeading___Toc362990544)

8.1. Study parameters/endpoints [17](#__RefHeading___Toc362990545)

8.1.1. Main study parameter/endpoint [17](#__RefHeading___Toc362990546)

8.1.2. Secondary study parameters/endpoints (if applicable) [17](#__RefHeading___Toc362990547)

8.1.3. Other study parameters (if applicable) [17](#__RefHeading___Toc362990548)

8.2. Randomisation, blinding and treatment allocation [17](#__RefHeading___Toc362990549)

8.3. Study procedures [17](#__RefHeading___Toc362990550)

8.4. Withdrawal of individual subjects [17](#__RefHeading___Toc362990551)

8.4.1. Specific criteria for withdrawal (if applicable) [17](#__RefHeading___Toc362990555)

8.5. Replacement of individual subjects after withdrawal [17](#__RefHeading___Toc362990556)

8.6. Follow-up of subjects withdrawn from treatment [18](#__RefHeading___Toc362990557)

8.7. Premature termination of the study [18](#__RefHeading___Toc362990558)

9. SAFETY REPORTING [19](#__RefHeading___Toc362990559)

9.1. Section 10 WMO event [19](#__RefHeading___Toc362990560)

9.2. AEs, SAEs and SUSARs [19](#__RefHeading___Toc362990561)

9.2.1. Adverse events (AEs) [19](#__RefHeading___Toc362990565)

9.2.2. Serious adverse events (SAEs) [19](#__RefHeading___Toc362990566)

9.2.3. Suspected Unexpected Serious Adverse Reactions (SUSAR's) [19](#__RefHeading___Toc362990567)

9.3. Annual safety report [19](#__RefHeading___Toc362990568)

9.4. Follow-up of adverse events [19](#__RefHeading___Toc362990569)

9.5. [Data Safety Monitoring Board (DSMB) / Safety Committee] [19](#__RefHeading___Toc362990570)

10. STATISTICAL ANALYSIS [20](#__RefHeading___Toc362990571)

10.1. Primary study parameter(s) [20](#__RefHeading___Toc362990572)

10.2. Secondary study parameter(s) [20](#__RefHeading___Toc362990573)

10.3. Other study parameters [20](#__RefHeading___Toc362990574)

10.4. Interim analysis (if applicable) [20](#__RefHeading___Toc362990575)

11. ETHICAL CONSIDERATIONS [21](#__RefHeading___Toc362990576)

11.1. Regulation statement [21](#__RefHeading___Toc362990577)

11.2. Recruitment and consent [21](#__RefHeading___Toc362990578)

11.3. Objection by minors or incapacitated subjects (if applicable) [21](#__RefHeading___Toc362990579)

11.4. Benefits and risk assessment, group relatedness [21](#__RefHeading___Toc362990580)

11.5. Compensation for injury [21](#__RefHeading___Toc362990581)

11.6. Incentives (if applicable) [21](#__RefHeading___Toc362990582)

12. ADMINISTRATIVE ASPECTS, MONITORING AND PUBLICATION [22](#__RefHeading___Toc362990583)

12.1. Handling and storage of data and documents [22](#__RefHeading___Toc362990584)

12.2. Monitoring and Quality Assurance [22](#__RefHeading___Toc362990585)

12.3. Amendments [22](#__RefHeading___Toc362990586)

12.4. Annual progress report [22](#__RefHeading___Toc362990587)

12.5. End of study report [22](#__RefHeading___Toc362990588)

12.6. Public disclosure and publication policy [22](#__RefHeading___Toc362990589)

13. STRUCTURED RISK ANALYSIS [23](#__RefHeading___Toc362990590)

13.1. Potential issues of concern [23](#__RefHeading___Toc362990591)

13.2. Synthesis [23](#__RefHeading___Toc362990592)

14. REFERENCES [24](#__RefHeading___Toc362990593)

**LIST OF ABBREVIATIONS AND RELEVANT DEFINITIONS**

| **ABR** | **ABR form, General Assessment and Registration form, is the application form that is required for submission to the accredited Ethics Committee (In Dutch, ABR = Algemene Beoordeling en Registratie)** |
| --- | --- |
| **AUC** | **Area under the Curve** |
| **BMI** | **Body Mass Index** |
| **CA** | **Competent Authority** |
| **CCMO** | **Central Committee on Research Involving Human Subjects; in Dutch: Centrale Commissie Mensgebonden Onderzoek** |
| **CV** | **Curriculum Vitae** |
| **EU** | **European Union** |
| **GCP** | **Good Clinical Practice** |
| **IC** | **Informed Consent** |
| **METC** | **Medical research ethics committee (MREC); in Dutch: medisch ethische toetsing commissie (METC)** |
| **PK** | **Pharmacokinetics** |
| **SPC** | **Summary of Product Characteristics (in Dutch: officiële productinfomatie IB1-tekst)** |
| **Sponsor** | **The sponsor is the party that commissions the organisation or performance of the research, for example a pharmaceutical**  **company, academic hospital, scientific organisation or investigator. A party that provides funding for a study but does not commission it is not regarded as the sponsor, but referred to as a subsidising party.** |
| **Wbp** | **Personal Data Protection Act (in Dutch: Wet Bescherming Persoonsgevens)** |
| **WMO** | **Medical Research Involving Human Subjects Act (in Dutch: Wet Medisch-wetenschappelijk Onderzoek met Mensen** |

**SUMMARY**

**Rationale:** Fentanyl is a strong opioïd and is highly lipophilic. Fentanyl pharmacokinetics are characterised by large inter- and inpatient differences, which may have serious consequences for the activity and toxicity profile of this drug. In this study we explore the influence of BMI and smoking behaviour on the pharmacokinetics (i.e. clearance (CL)) of fentanyl.

**Objective:** Primary objective: To study the relation between BMI and the pharmacokinetics of fentanyl, in patients using a stable dose of the fentanyl patch (Durogesic ®). Secundary objective: to study the relation between smoking and the pharmacokinetics of fentanyl, in patients using a stable dose of the fentanyl patch (Durogesic ®).

**Study design:** explorative cohort study

**Study population:** All patients using a stable dose of a fentanyl (Durogesic ®) patch (for at least 8 days).

**Intervention (if applicable)**: not applicable

**Main study parameters/endpoints:** Pharmacokinetics (Clearance, AUC, etc.)

**Nature and extent of the burden and risks associated with participation, benefit and group relatedness:** This study has an **extremely low** risk for the participating patients. After taking 1 blood sample, the study is finished. There is no direct benefit for the patient.

# INTRODUCTION AND RATIONALE

Pain is a common problem in cancer patients, occurring both in curative settings as well as in palliative settings. Opioids are often used to treat cancer pain. Fentanyl is one of the most widely used opioids.

Several preparations of fentanyl are currently available . The transdermal fentanyl patch is developed for maintenance medication in chronic pain and is the most commonly used form. Patches are available in different sizes, consistent with specific delivery doses of these patches (12ug/hr, 25ug/hr, 50ug/hr, 75ug/hr or 100ug/hr). Fentanyl is absorbed through the intact skin and a constant dose of drug is absorbed. Two different patches have been developed; a reservoir patch and a matrix patch. These patches differ in the way fentanyl is stored. However farmacokinetically, they are comparable . Nowadays, only the matrix patch is used in clinical practice.

After placement of the patch, the plasma fentanyl concentration gradually increases. After 72hrs the patch has to be changed for a new one, as a stable diffusion of the drug through the skin is no longer guaranteed. Steady state concentrations are approached when a second transdermal fentanyl patch is sticked on the skin . Removing the patch will not immediately lead to diminished fentanyl concentrations because of the amount of fentanyl stored in subcutaneous depots, and therefore systemic concentrations will gradually decrease.

Fentanyl is a drug that is highly lipophilic and binds strongly to plasma proteins. Fentanyl is more potent than morphine at equipotent dose levels . The metabolism of fentanyl takes place primarily in the liver . Fentanyl is mainly oxidized into the inactive metabolite norfentanyl by the CYP3A4 iso-enzyme . Less than 1% is metabolized to despropionyl-fentanyl, hydroxyfentanyl, and hydroxynorfentanyl, which are also inactive metabolites. Fentanyl is mainly excreted renally and for a minor part through the feces. The large majority of the fentanyl is excreted as the metabolites mentioned; 10% as unchanged drug .

Unfortunately there is a wide pharmacokinetic intra- and interpatient variability in patients using a fentanyl patch. It is largely unclear which factors contribute to this variability . It is crucial to know which factors influence fentanyl concentrations because of the risk of over- and underdosing of fentanyl. An overdose of fentanyl could lead to serious complications, including respiratory depression or ultimately death, while underdosing fentanyl may lead to inadequate pain relief.

Fentanyl is dosed by titration. When pain is inadequately treated and side effects are manageable the fentanyl dose is usually increased. Dose finding by titration cannot be used in patients who switch from another opioid to fentanyl. Usually these patients start with more or less the equi-analgetic dose of fentanyl. Especially in these cases it would be extremely helpful if could be predicted if these patients are at risk for under- or overdosing of fentanyl.

Fentanyl is highly liphophilic and will be absorbed by the subcutaneous fat-tissue. We hypothesize that higher fentanyl concentrations will be reached when the patch is used by patients with thicker subcutaneous fat layers, represented by patients with a higher body mass index (BMI). Most farmacokinetic studies with fentanyl are performed in healthy volunteers or in patients undergoing elective surgery. Unfortunately, not all studies reported the BMI of the included patients. In studies with healthy volunteers BMI was under 30kg/m2 or mean weight between 70 and 80 kg . Most studies in patients undergoing surgery only included patients with a weight under 100kg . Just a few studies have studied fentanyl farmacokinetics in cancer patients. One of these studies showed significantly lower fentanyl concentrations in cachectic patients (mean BMI 16 kg/m2) than in normal weigth patients (mean BMI 23 kg/m2) using a fentanyl patch for 48 - 72hr . Two other farmacokinetic studies in cancer patients did not show significant differences between normal weight patients compared to cachectic patients . However, in these studies patients in the lowest BMI group had a BMI ≤ 18.5 instead of a BMI of 16 in the study of Heiskanen .

Another factor that can be of influence on the pharmacokinetics of medication is cigarette smoking. As 25% of the population in the Netherlands smokes cigarettes, male vs female is 27% -23 %, probably a significant part of the fentanyl users also are smokers .. .

The polycyclic aromatic hydrocarbons in cigarette smoke are believed to be responsible

for the induction of cytochrome P450.. A study with erlotinib showed a significantly decreased AUC in smokers compared to nonsmokers . Both erlotinib and fentanyl are mostly metabolized by CYP 3A4. A small part of the metabolism of erlotinib is also influenced by CYP 1A1. Cigarette smoke induces CYP 1A1 and can lead to a cascade, which also involves CYP 3A4, thereby influencing the clearance of erlotinib . Probably, it also influences the metabolism of fentanyl. A study from our group with irinotecan showed a lower exposure to SN-38 (the active irinotecan metabolite) in smokers compared to non-smokers. The hypothesis was that irinotecan is highly sensitive to CYP3A induction and that this is modulated by cigarette smoking .. Possible influence of smoking on clearance of fentanyl is important to know. Terminally ill patients often change their habits in the last part of life. Heavy smokers are physically not able to smoke as much as they did before, or occasional smokers smoke more because of stress. Both scenarios can potentially influence the fentanyl clearance. This may result in over- or underdosing of fentanyl, causing intoxication or ineffective pain treatment.

As a result of these earlier publications, in this study we want to determine which patient characteristics influence the pharmacokinetics of fentanyl.

# OBJECTIVES

Primary Objective:

- to study the effect of BMI on pharmacokinetics of fentanyl by comparing the AUC between patients with a BMI < 20 and patients with a a BMI > 25, both using a stable dose of the fentanyl patch (Durogesic ®).

Secundary Objective:

- to study the effect of smoking on pharmacokinetics of fentanyl by comparing the AUC between smokers and non-smokers, both using a stable dose of the fentanyl patch (Durogesic ®).

Stable dose is defined as using the same dose of fentanyl during at least 8 days.

# STUDY DESIGN

This is a single-center pharmacokinetic cohort study. The trial will be performed at the Erasmus MC Cancer Institute, department of Medical Oncology.

Patients with a stable dosage of fentanyl used through a patch can be included. Stable dose is defined as at least 8 days using a stable fentanyl dosage. Blood samples are always taken on the 2^nd^ day of the used patch.

Patients will be asked to stick a fentanyl patch (Durogesic ®) during 3 periods at the upper arm. During the third period, a venous blood sample will be taken. This blood sample will be taken at the same moment as a regular blood sampling is performed.

After taking the blood sample the patient has finished the study.

# STUDY POPULATION

## Population

Patients for the study will be recruited at the outpatient department of Medical Oncology in both locations of the Erasmus MC Cancer Institute.

## Inclusion criteria

In order to be eligible to participate in this study, a subject must meet all of the following criteria:

- age ≥ 18 years;
- stable use and dose of fentanyl patch (Durogesic ®) for at least 8 days, irrespective of the dose used;
- written informed consent.

## Exclusion criteria

A potential subject who meets any of the following criteria will be excluded from participation in this study:

- Using fentanyl as rescue medication (other opioids are allowed)
- Serious psychiatric illness, confusion of intellectual disability
- The use of strong cytochrome P450 inhibitors or inducers.

## Sample size calculation

BMI

With a suggested inter-patient variability in fentanyl pharmacokinetics of 25%, a power of 0.8 and two-side testing (P <0.025) at least **20 evaluable patients** in each group are required to determine a difference in exposure between the two BMI-groups of 25%. To evaluate the differences in plasma fentanyl concentrations the Student’s t test is used. Smokers and non-smokers will be distributed equally in these BMI-groups.

Smoking

Around 25% of the Dutch population smokes. In this study at least 20 patients have to be smokers and at least 20 have to be non-smokers to detect a similar difference in exposure of 25%. Patients can be included independently of their BMI. Smokers are patients who are smoking daily any kind of tobacco or e-cigarettes. Non-smokers are patients who quit smoking at least one month before PK sampling.

In conclusion, the cohort exists of:

- At least 20 patients with BMI < 20 (about 5 smokers)
- At least 20 patients with BMI > 25 (about 5 smokers)
- At least 20 patients defined as smokers
- At least 20 patients defined as non-smokers
- Dependent on the number of smokers in the extreme BMI groups: about 40 patients with

20 ≤ BMI ≤ 25 (about 10 smokers)

In total we will include a maximum of 80 patients.

# TREATMENT OF SUBJECTS

Not applicable

## Investigational product/treatment

## Use of co-intervention (if applicable)

## Escape medication (if applicable)

# INVESTIGATIONAL PRODUCT

Not applicable.

## Name and description of investigational product(s)

## Summary of findings from non-clinical studies

## Summary of findings from clinical studies

## Summary of known and potential risks and benefits

## Description and justification of route of administration and dosage

## Dosages, dosage modifications and method of administration

## Preparation and labelling of Investigational Medicinal Product

## Drug accountability

# NON-INVESTIGATIONAL PRODUCT

Not applicable

## Name and description of non-investigational product(s)

## Summary of findings from non-clinical studies

## Summary of findings from clinical studies

## Summary of known and potential risks and benefits

## Description and justification of route of administration and dosage

## Dosages, dosage modifications and method of administration

## Preparation and labelling of Non Investigational Medicinal Product

## Drug accountability

# METHODS

## Study parameters/endpoints

Farmacokinetic parameters (i.e. clearance, AUC)

### Main study parameter/endpoint

Farmacokinetic parameters (i.e. clearance, AUC)

### Secondary study parameters/endpoints (if applicable)

Not applicable

### Other study parameters (if applicable)

Not applicable

## Randomisation, blinding and treatment allocation

Not applicable

## Study procedures

Pharmacokinetic sampling

Fentanyl pharmacokinetics will be performed with a single blood sample during stable dosing.. One blood sample (4.5 mL) will be drawn through a cannula from a vein in the arm (**opposite to the one where the fentanyl patch is placed**). The blood sample will be collected in the presence of potassium EDTA as anticoagulant and will be processed to plasma within 10 minutes by centrifugation for 10 min at 2,500-3,000*g at 4°C. Plasma will be transferred into polypropylene tubes (1.8 mL Nunc vials), which will be stored at T<-70°C (T<-20°C during collection period) until time of analysis.

Pharmacokinetic data analysis

All fentanyl drug concentrations will be measured at the Laboratory of Translational Pharmacology of the Erasmus MC, Rotterdam by the use of a validated mass-spectometry (LC-MS/MS) assay.

Pharmacokinetic data analysis will take place using WinNonlin or other an appropriate method to calculate exposure measures (i.e. CL) at the Laboratory of Translational Pharmacology of the Erasmus MC, Rotterdam.

## Withdrawal of individual subjects

Subjects can leave the study at any time for any reason if they wish to do so without any consequences. The investigator can decide to withdraw a subject from the study for urgent medical reasons.

### Specific criteria for withdrawal (if applicable)

Not applicable

## Replacement of individual subjects after withdrawal

In this study we will include a maximum of 80 evaluable patients (due to a maximum of financial recourses).

## Follow-up of subjects withdrawn from treatment

Not applicable

## Premature termination of the study

Not applicable.

# SAFETY REPORTING

Not applicable

## Section 10 WMO event

## AEs, SAEs and SUSARs

### Adverse events (AEs)

### Serious adverse events (SAEs)

### Suspected Unexpected Serious Adverse Reactions (SUSAR's)

## Annual safety report

## Follow-up of adverse events

## [Data Safety Monitoring Board (DSMB) / Safety Committee]

Not applicable.

# STATISTICAL ANALYSIS

The clearance of all different samples is calculated. This is independent of the dose because of linear pharmacokinetics. After this calculation we will use the Student’s t test to compare the clearance for fentanyl in patients with a BMI < 20 compared to a BMI > 25 and for smokers versus non-smokers.

For all 4 of these groups at least 20 patients have to be included.

## Primary study parameter(s)

Fentanyl pharmacokinetics (i.e. clearance).

## Secondary study parameter(s)

Not applicable.

## Other study parameters

Not applicable.

## Interim analysis (if applicable)

Not applicable.

# ETHICAL CONSIDERATIONS

## Regulation statement

This study will be performed in accordance with the Declaration of Helsinki as adopted by the 18th WMA General Assembly, Helsinki, Finland, June 1964 and amended by the: 29th WMA General Assembly, Tokyo, Japan, October 1975, 35th WMA General Assembly, Venice, Italy, October 1983, 41st WMA General Assembly, Hong Kong, September 1989, 48th WMA General Assembly, Somerset West, South Africa, October 1996, 52nd WMA General Assembly, Edinburgh, Scotland, October 2000, 53rd WMA General Assembly, Washington, DC, USA, October 2002(Note of Clarification on paragraph 29 added), 55th WMA General Assembly, Tokyo, Japan, October 2004 (Note of Clarification on Paragraph 30 added), 59th WMA General Assembly, Seoul, Korea, October 2008. (appendix).

## Recruitment and consent

Subjects will be informed about the study by the investigator, doctor, research nurse or nurse practioner.

## Objection by minors or incapacitated subjects (if applicable)

Not applicable

## Benefits and risk assessment, group relatedness

Not applicable

## Compensation for injury

The sponsor/investigator has a liability insurance which is in accordance with article 7, subsection 6 of the WMO.

## Incentives (if applicable)

Not applicable

# ADMINISTRATIVE ASPECTS, MONITORING AND PUBLICATION

## Handling and storage of data and documents

Data are handled by the investigator and his team. Data are coded by a number independently of birthdate or initials. The key to the code is safeguarded by the head investigator. After the study the coded data will be saved for 15 years

## Monitoring and Quality Assurance

This study is a (extremely) low risk study. Monitoring consists of checking the first 3 informed consent forms followed by checking randomly 10% of the informed consent forms.

## Amendments

Not applicable

## Annual progress report

Not applicable

## End of study report

Not applicable

## Public disclosure and publication policy

As results of this study will be presented/published. All investigators mentioned will be part of the publications.

# STRUCTURED RISK ANALYSIS

Not applicable

## Potential issues of concern

## Synthesis

# REFERENCES

**1. Kuip EJM, Zandvliet ML, van der Rijt CCD, Mathijssen RHJ. Pharmacological and clinical aspects of immediate release fentanyl preparations: criteria for selection. European Journal of Hospital Pharmacy. 2012.**

**2. Marier JF, Lor M, Morin J, Roux L, Di Marco M, Morelli G, et al. Comparative bioequivalence study between a novel matrix transdermal delivery system of fentanyl and a commercially available reservoir formulation. British Journal of Clinical Pharmacology. 2007 Jan;63(1):121-4.**

**3. Kress HG, Boss H, Delvin T, Lahu G, Lophaven S, Marx M, et al. Transdermal fentanyl matrix patches Matrifen and Durogesic DTrans are bioequivalent. European Journal of Pharmaceutics & Biopharmaceutics. 2010 Jun;75(2):225-31.**

**4. Portenoy RK, Southam MA, Gupta SK, Lapin J, Layman M, Inturrisi CE, et al. Transdermal fentanyl for cancer pain. Repeated dose pharmacokinetics. Anesthesiology. 1993 Jan;78(1):36-43.**

**5. Helm S, Trescot AM, Colson J, Sehgal N, Silverman S. Opioid antagonists, partial agonists, and agonists/antagonists: the role of office-based detoxification. Pain Physician. 2008 Mar-Apr;11(2):225-35.**

**6. Smith HS. Opioid metabolism. Mayo Clinic Proceedings. 2009 Jul;84(7):613-24.**

**7. Feierman DE, Lasker JM. Metabolism of fentanyl, a synthetic opioid analgesic, by human liver microsomes. Role of CYP3A4. Drug Metabolism & Disposition. 1996 Sep;24(9):932-9.**

**8. Van Nimmen NF, Poels KL, Menten JJ, Godderis L, Veulemans HA. Fentanyl transdermal absorption linked to pharmacokinetic characteristics in patients undergoing palliative care. Journal of Clinical Pharmacology. 2010 Jun;50(6):667-78.**

**9. Solassol I, Caumette L, Bressolle F, Garcia F, Thezenas S, Astre C, et al. Inter- and intra-individual variability in transdermal fentanyl absorption in cancer pain patients. Oncology Reports. 2005 Oct;14(4):1029-36.**

**10. Marier JF, Lor M, Potvin D, Dimarco M, Morelli G, Saedder EA. Pharmacokinetics, tolerability, and performance of a novel matrix transdermal delivery system of fentanyl relative to the commercially available reservoir formulation in healthy subjects. Journal of Clinical Pharmacology. 2006 Jun;46(6):642-53.**

**11. Moore KT, Adams HD, Natarajan J, Ariyawansa J, Richards HM. Bioequivalence and safety of a novel fentanyl transdermal matrix system compared with a transdermal reservoir system. Journal of Opioid Management. 2011 Mar-Apr;7(2):99-107.**

**12. Sathyan G, Guo C, Sivakumar K, Gidwani S, Gupta S. Evaluation of the bioequivalence of two transdermal fentanyl systems following single and repeat applications. Curr Med Res Opin. 2005 Dec;21(12):1961-8.**

**13. Duthie DJ, Rowbotham DJ, Wyld R, Henderson PD, Nimmo WS. Plasma fentanyl concentrations during transdermal delivery of fentanyl to surgical patients. British Journal of Anaesthesia. 1988 May;60(6):614-8.**

**14. Plezia PM, Kramer TH, Linford J, Hameroff SR. Transdermal fentanyl: pharmacokinetics and preliminary clinical evaluation. Pharmacotherapy. 1989;9(1):2-9.**

**15. Sandler AN, Baxter AD, Katz J, Samson B, Friedlander M, Norman P, et al. A double-blind, placebo-controlled trial of transdermal fentanyl after abdominal hysterectomy. Analgesic, respiratory, and pharmacokinetic effects. Anesthesiology. 1994 Nov;81(5):1169-80; discussion 26A.**

**16. Varvel JR, Shafer SL, Hwang SS, Coen PA, Stanski DR. Absorption characteristics of transdermally administered fentanyl. Anesthesiology. 1989 Jun;70(6):928-34.**

**17. Heiskanen T, Matzke S, Haakana S, Gergov M, Vuori E, Kalso E. Transdermal fentanyl in cachectic cancer patients. Pain. 2009 Jul;144(1-2):218-22.**

**18. stivoro.nl.**

**19. Lewis LD, Ratain MJ. Might cigarettes be a "smoking gun" to reduce taxane myelotoxicity? Clinical Cancer Research. 2012 Aug 15;18(16):4219-21.**

**20. Zevin S, Benowitz NL. Drug interactions with tobacco smoking. An update. Clinical Pharmacokinetics. 1999 Jun;36(6):425-38.**

**21. Hamilton M, Wolf JL, Rusk J, Beard SE, Clark GM, Witt K, et al. Effects of smoking on the pharmacokinetics of erlotinib. Clinical Cancer Research. 2006 Apr 1;12(7 Pt 1):2166-71.**

**22. van der Bol JM, Mathijssen RH, Loos WJ, Friberg LE, van Schaik RH, de Jonge MJ, et al. Cigarette smoking and irinotecan treatment: pharmacokinetic interaction and effects on neutropenia. Journal of Clinical Oncology. 2007 Jul 1;25(19):2719-26.**

**23. Gourlay GK, Kowalski SR, Plummer JL, Cherry DA, Gaukroger P, Cousins MJ. The transdermal administration of fentanyl in the treatment of postoperative pain: pharmacokinetics and pharmacodynamic effects. Pain. 1989 May;37(2):193-202.**
